# The microstructure and the origin of the Venus from Willendorf

**DOI:** 10.1038/s41598-022-06799-z

**Published:** 2022-02-28

**Authors:** Gerhard W. Weber, Alexander Lukeneder, Mathias Harzhauser, Philipp Mitteroecker, Lisa Wurm, Lisa-Maria Hollaus, Sarah Kainz, Fabian Haack, Walpurga Antl-Weiser, Anton Kern

**Affiliations:** 1 Department of Evolutionary Anthropology & Core Facility for Micro-Computed Tomography, University of Vienna, Djerassiplatz 1, 1030 Vienna, Austria; 2 Geological-Paleontological Department, Natural History Museum Vienna, Vienna, Austria; 3 Department of Evolutionary Biology, University of Vienna, Vienna, Austria; 4 Clinic of Small Animal Surgery and Reproduction, Ludwig-Maximilians University, Munich, Germany; 5 Württemberg State Museum Stuttgart, Stuttgart, Germany; 6 Department of Prehistory, Natural History Museum Vienna, Vienna, Austria; 7 Human Evolution and Archaeological Sciences-HEAS, University of Vienna, Vienna, Austria

**Keywords:** Anthropology, Archaeology

## Abstract

The origin and key details of the making of the ~ 30,000 year old Venus from Willendorf remained a secret since its discovery for more than a hundred years. Based on new micro-computed tomography scans with a resolution of 11.5 µm, our analyses can explain the origin as well as the choice of material and particular surface features. It allowed the identification of internal structure properties and a chronological assignment of the Venus oolite to the Mesozoic. Sampling numerous oolite occurrences ranging ~ 2500 km from France to the Ukraine, we found a strikingly close match for grain size distribution near Lake Garda in the Southern Alps (Italy). This might indicate considerable mobility of Gravettian people and long-time transport of artefacts from South to North by modern human groups before the Last Glacial Maximum.

## Introduction

The Venus I figurine was found on the left bank of the Danube in Willendorf II/Lower Austria on August 7th, 1908^1,2^ during excavations led by Josef Szombathy, supervised by Hugo Obermaier and Josef Bayer. The excavation documentation leaves space for interpretation as far as the attribution of the horizon with the figurine to layer Willendorf II/9 (29.1–28.8 ka cal BP^3^) is concerned, but the figurine evidently comes from an archaeological horizon 25 cm below^2^ Layer 9, possibly equivalent with Layer 8a (< 30.8–29.2 ka cal BP^3^), and is therefore roughly 30,000 years old. Both layers are associated with Gravettian industries. The occupation of Willendorf II itself goes far back into the Early Aurignacian, when people first settled in a cold steppe-type environment 43,500 years BP^3^. Willendorf is thus one of the earliest evidences in Europe for early modern human settlings and emphasizes the significant role of the Danube corridor for modern human dispersal.

**Figure 1:**
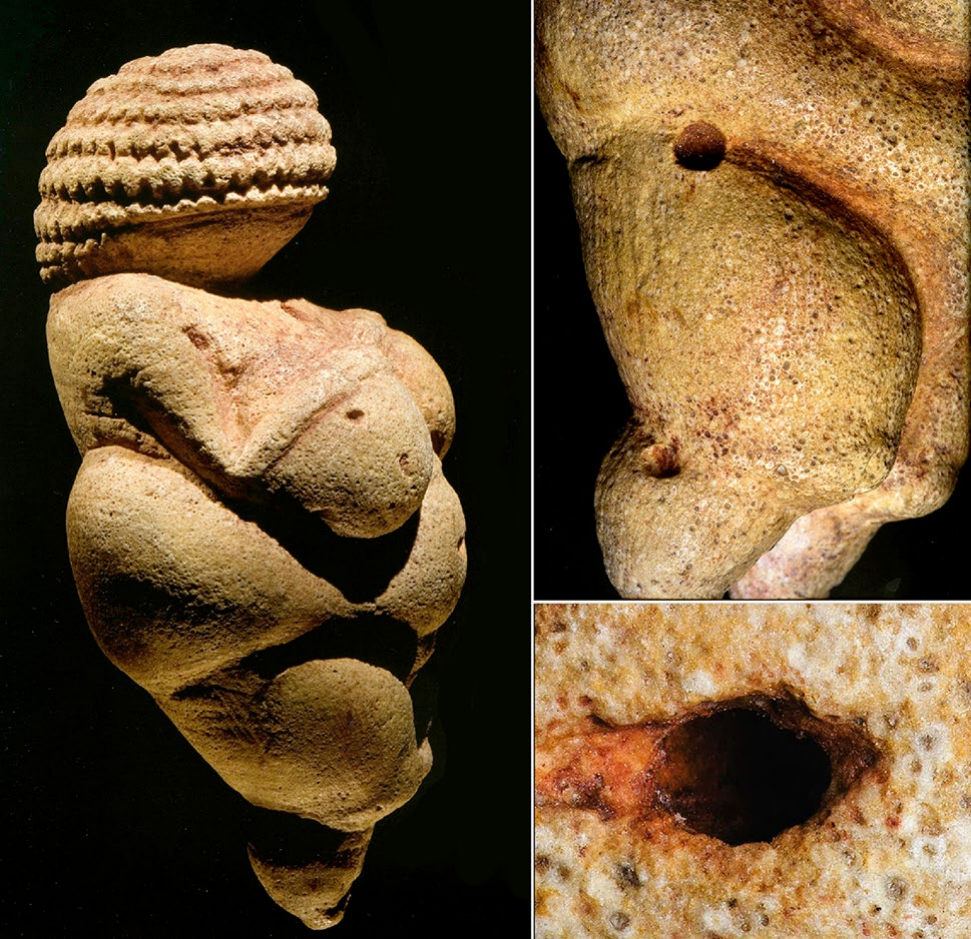
The original Venus from Willendorf. Left: lateral view. Right-top: hemispherical cavities on the right haunch and leg. Right bottom: existing hole enlarged to form the navel. All images by Lois Lammerhuber^4^.

The statuette (Fig. 1), reposited at the Natural History Museum in Vienna, is exactly 110 mm in height and represents a symbolized adult and faceless female with exaggerated genitalia, pronounced haunches, a protruding belly, heavy breasts, and a sophisticated headdress or hairdo^4,5^. The figurine was made from oolitic limestone and painted red, possibly with ochre, which was almost entirely removed by cleaning at the time of discovery. Oolitic limestones are completely absent in and around Willendorf. The origin of the raw material was elusively discussed earlier^6^, and already the discoverer Josef Szombathy suspected that the raw material of the Venus was collected elsewhere, potentially in the nearby Vienna Basin where Tertiary limestone deposits exist. In the last years, Stránská Skála (Czech Republic) was conferred to represent the potential source of material for the Venus from Willendorf^7^.

The figurines from Willendorf (alongside the Venus I made from oolitic limestone, there are two younger ivory figurines: Venus II and III) are part of a system of representations distributed from France to Russia, and are very similar to different Russian figurines. Based on shape and making, a connection to the Ukraine/South Russia has been claimed, placing figurines from Pavlov-Willendorf-Kostenki-Avdeevo in one cluster^8^. Among the female representations of the Gravettian there are supraregional types such as the naturalistic figurines, e. g., Lespugue, Willendorf and Kostenki, and abstract representations often combining male and female characteristics, also distributed from France to Russia. Types like the figurines from Renancourt in Northern France^9^, the Mediterranean figurines or the figurines from Mal’ta in Siberia^10^ are bound to the respective regions. Chronologically, the Venus I from Willendorf is slightly older than the figurines from Eastern Europe and also those from France. The youngest group of sculptures are the Balzi Rossi figurines from Italy^11^. Many figurines from France and Italy are not stratified and can therefore not be dated. Older female figurines such as the Venus from Hohle Fels^12^—stylistically closely related to other Aurignacian figurines—are commonly regarded as a link to the female representations of the Gravettian.

**Figure 2:**
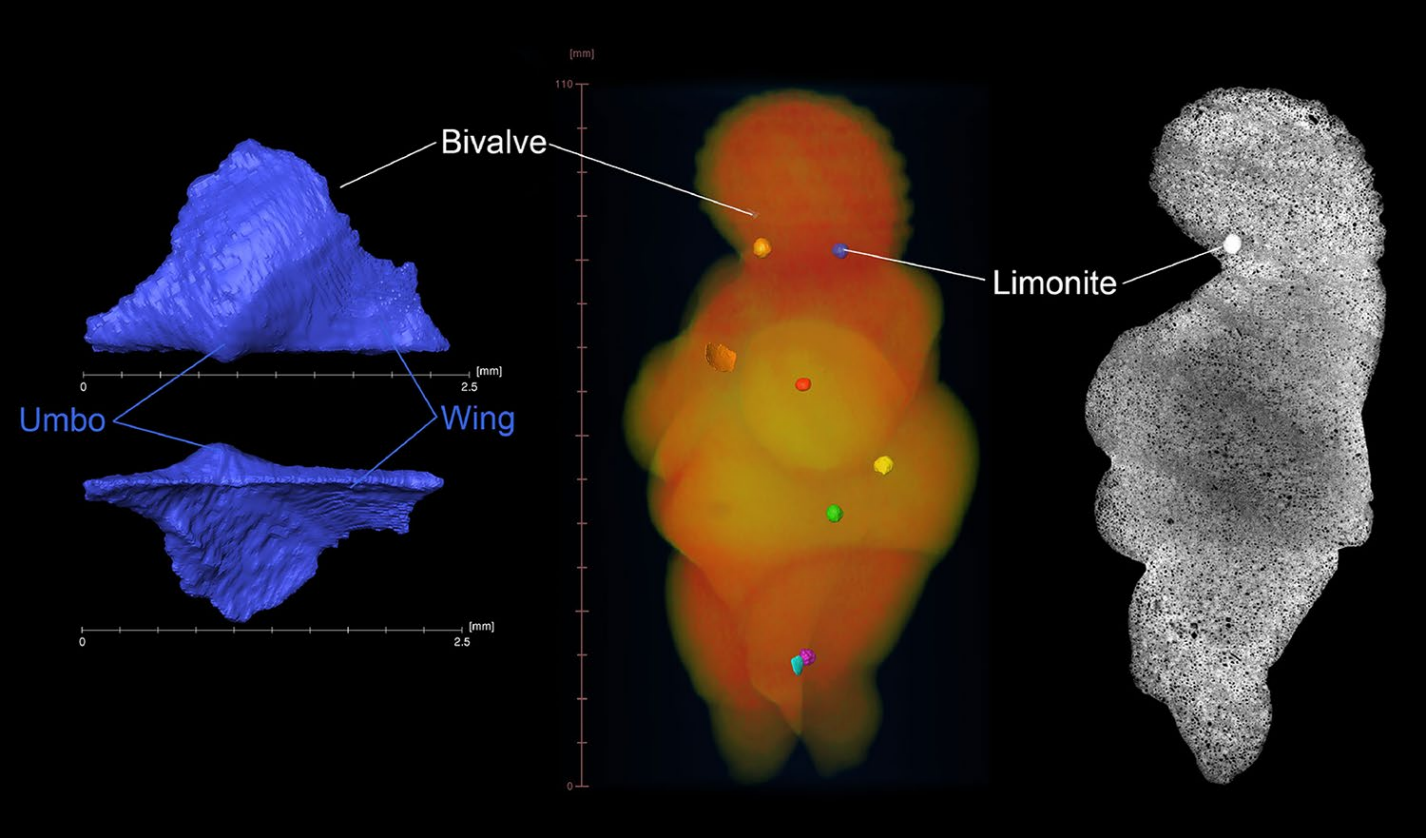
Pictures derived from µCT scans of the Venus. Left: Segmented bivalve (*Oxytomidae*) that was located on the right side of the Venus head; scan resolution 11.5 µm; characteristic features are the umbo and the wings. Middle: Volume rendering of the virtual Venus; six embedded limonite concretions: neck right (orange), neck left (blue), breast left (red), belly left (yellow), hip left (green), leg left (purple); three mollusc fragments: bivalve head right (blue, only 2.5 mm long, see white line from label “Bivalve” for position), shell breast middle (orange), shell leg left (turquoise). Right: Single µCT-slice showing the porosity and layering of the oolite; note the relative density of the limonite concretion; scan resolution 53 µm.

Because of the unique value of the Venus from Willendorf, one of the most famous signs of early modern human symbolic behaviour, invasive investigations have been impossible since its discovery in 1908. The availability of micro-computed tomography (µCT; Fig. 2, Extended Data Fig. 1) provided the first chance to radiograph the figurine in 3D in a resolution close to thin-sections and microscopy, which paved the way to also explore the interior of the raw material. The Venus was first scanned on January 8th 2013 with a spatial resolution of 53 µm (Fig. 2; see “Methods”). These data already hinted at the heterogenous internal structure, showing the inclusions of biogenic material and other larger particles as well as the stratification of the layered sediment and the dissolution of ooid nuclei. On March 22nd 2016 further scans with a resolution of 11.5 µm were undertaken with a focus on the leg and the head regions (Fig. 2, see “Methods”).

## Results

The 3D analyses of the µCT data revealed that the rock represents an oomouldic limestone, consisting of calcitized ooids with layered, micritic cortex. The leaching of the ooid-nuclei is a result of post-sedimentary meteoric leaching of originally aragonitic ooids^13^. The nature of the original nuclei is unknown but very likely was formed by aragonite particles, and not by quartz sand, which would have resisted leaching. The internal crossbedding is indicated by layers of smaller oomoulds alternating with layers of slightly larger oomoulds (Fig. 2). Within these layers, the particles are moderately well sorted and grain-supported. The ooid-diameters were measured from µCT cross-sections, which are comparable to 2D petrographic thin-sections. The resulting object-size distributions are not reflecting the real grain-size distribution within the rock but capture various cutting levels of the ooids. The resulting mean object size in the Venus is 0.253 mm (S.D. = 0.131 mm, max. = 1.289, min. = 0.036 mm). Six sub-spherical iron-oxide concretions of 2.77 mm mean diameter are randomly distributed within the volume of the figurine (Fig. 2). Fossils are frequent within the rock. Scattered shell-fragments of bivalves appear in the scan as cavities due to dissolution of the original shells. Although the surfaces of the cavities still reflect the original morphology of the fragments, most objects lack morphological features that would allow any closer identification.

Nevertheless, a few larger shell fragments represent initial parts (umbones) of bivalves. Due to the curvature, this part of the bivalve shell is more resistant to mechanical breakage in agitated environments. The umbo is associated with wing-like structures and only weakly protruding over the straight dorsal margin; no hinge teeth are developed (Fig. 2). This morphology is characteristic of members of the bivalve families *Bakevelliidae, Pteriidae* and *Oxytomidae*^14^ (see Extended Data Fig. 2). Tentatively, we consider the fragment in the Venus to represent an *Oxytomidae*, based on comparisons with *Oxytoma* specimens in the collections of the Natural History Museum Vienna. *Oxytomidae* have a Permian to Cretaceous distribution^14^, but only the Mesozoic representatives agree in morphology with the fragment preserved in the Venus. The fossils suggest a Mesozoic age of the Venus oolite (251–66 Million years ago) and exclude an origin from Miocene (23–5.3 Million years ago) oolites. This dating would also be supported if the fragments were *Bakevellidae*, as this family originated in the Permian and became extinct during the Eocene^14^. *Pteriidae*, by contrast, appeared during Triassic times and persist until today^14^. This family, however, became extirpated in the Miocene Sarmatian Sea at around 12.8 Ma during the Badenian-Sarmatian Extinction Event^15^ and does not occur in any Sarmatian oolites. An origin of the Venus material from Miocene oolites of the Vienna Basin can thus be excluded.

The surface of the Venus exhibits several hemispherical cavities (see Fig. 1), among them five larger ones in the décolleté region, at the right haunch, the lateral right knee, the backside of the left thigh, and the navel. Some smaller pits are found on the right breast, in the face region, and the left upper arm (Extended Data Table 2). In earlier investigations, there were speculations as to whether the navel was a natural pit or made intentionally^5^. The nature of the other pits was inexplicable since they do not correspond to any anatomical features. In the light of the now discovered limonite inclusions there is a straightforward explanation available. The nearly spherical limonite concretions are scattered randomly throughout the rock and are much more resistant than the circumjacent oolite material. During the carving process, the creator would have had to deal with these disturbing elements, which would have frequently broken off due to processing. Based on its size, the cavity at the navel could indeed have resulted from a limonite concretion which was broken off and was turned into a feature. It is also possible that the navel limonite concretion has been removed intentionally since we could observe a larger elongated furrow right and a smaller furrow left to the navel, which are angled downwards into the pit (Fig. 1). Measurements of diameters in all three dimensions of the six embedded limonite concretions (they are not perfectly spherical) resulted in a mean of 2.77 mm, which is very similar to the mean of 2.57 mm for the eight hemispherical cavities on the figurine’s surface (Extended Data Table 2), which supports the conclusion that the cavities on the surface result from broken-off limonite concretions. Reflected-light microscopy (Extended Data Fig. 3) on the original Venus also demonstrated that the dissolution of nuclei occurred prior to the carving of the figurine, because the ooid nuclei are also missing on the surface and the cutting edges are blurred. This is exactly what one would expect if the thin, hollow, coreless ooid shells had been cut by a burin. The fact that anthropogenic coloring of the figurine fills the cavities of the oomoulds on the surface of the Venus further supports this notion. The leaching of the oolite is potentially the major reason why this raw material was selected. While an oolite with still embedded nuclei would be highly compact, more difficult to handle and heavier, the oolite used for the Venus has different material properties. Due to the dissolved nuclei, the material is porous and compares to today’s foam-concrete, offering much easier conditions for the production process and lighter weight for transportation.

One of the most intriguing open questions with regard to the Venus is where the figurine, or at least its raw material, originates. We considered the distribution of ooid diameters measured from non-invasive µCT scans (Venus from Willendorf, Pendant from Brillenhöhle) and invasive thin-sections (all other localities) for statistical comparison. European oolite deposits between France in the West and the Ukraine and Crimea in the East, from Germany in the North to Sicily in the South were included (Fig. 4, Extended Data Table 1).

**Figure 3:**
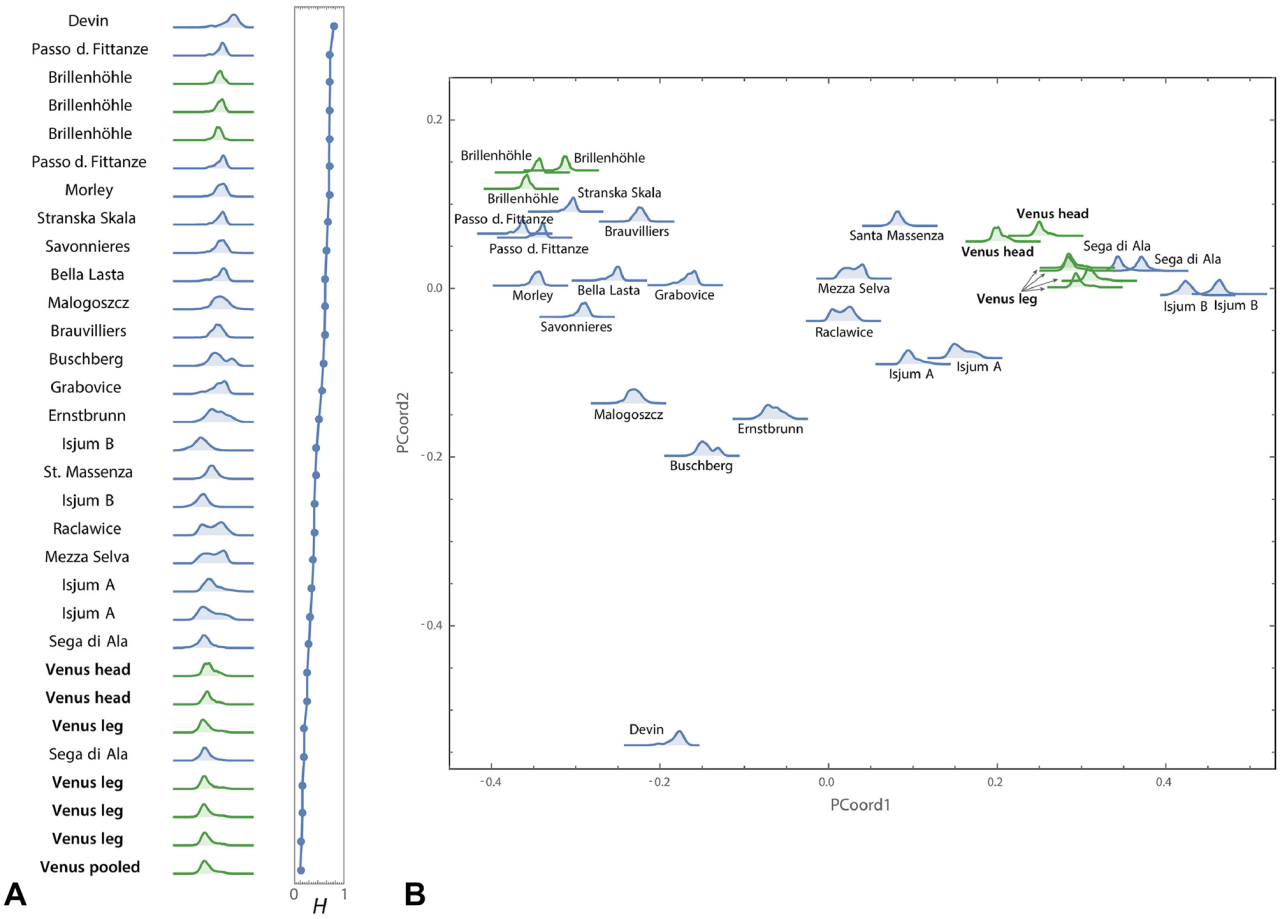
Comparison of the grain size distribution of the investigated oolite sites (archaeological material in green). Left: Hellinger distances measuring the overlap of two distributions, where *H* = 0 indicates identical distributions and *H* = 1 no overlap at all (see “Methods”). Right: Ordination analysis (principal coordinate analysis) of all pairwise Hellinger distances between sites. The first two principal coordinates accounted for 76% of the variation in Hellinger distances.

**Figure 4:**
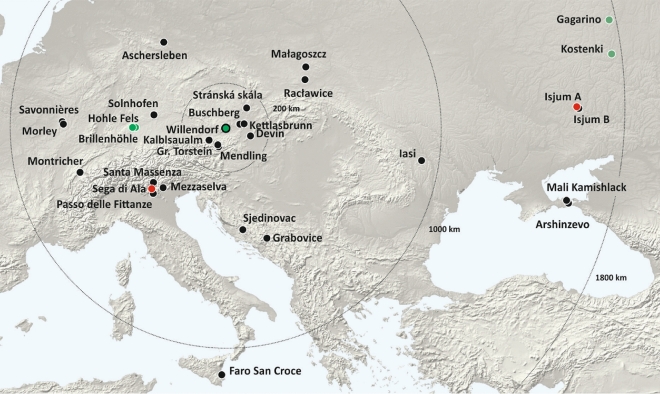
Map of sampled oolite localities (black); the two most likely sites of origin Sega di Ala and Isjum A are indicated in red. The archaeological sites Willendorf, Brillenhöhle, Hohle Fels, Kostenki, and Gagarino are shown in green. The digital elevation model was created with ArcGIS 10.4 and is based on^18^, which was obtained from the WorldClim database. https://desktop.arcgis.com/de/arcmap/10.4/get-started/setup/arcgis-desktop-quick-start-guide.htm.

Prior to statistical examination, we excluded all localities (Extended Data Table 4) exhibiting characteristics that were incompatible with the Venus material with regard to age (e.g., all Miocene sites) or sedimentary composition (e.g., differing fundamentally from the Venus by a very high number of bioclasts). For these remaining localities, we quantified the overall dissimilarity of the statistical distributions of the ooid size measurements by the Hellinger distances (see “Methods”). The six different samples from the Venus’ head and leg regions show the smallest Hellinger distances to each other and cluster closely together in the principal coordinate analysis (Fig. 3A,B, Extended Data Table 3). One other locality, Sega di Ala (located in a side valley of Lake Garda, Italy), is indistinguishable from this Venus cluster (Fig. 3A, Fig. 4). At first, we had only one sample from this site, which intermingled perfectly with the Venus samples. To validate this finding, we sampled and measured a second thin section from Sega di Ala, which confirmed the close resemblance between the Venus material and the samples from Sega di Ala (see Extended Data Table 3). Likewise, we included another thin section from Isjum (eastern Ukraine), which was the second most likely candidate in the first analyses, and after inclusion of a further thin section remained second (Extended Data Table 3). Beside the Venus from Willendorf, there was another archaeological object in our sample. As aforementioned, no other female palaeolithic statuette made from oolite is known so far. However, the Brillenhöhle in southwest Germany^16^, near the famous site of Hohle Fels, yielded a fragment of a personal ornament made of oolitic limestone in Layer VII, dated to approximately 32–31 ka cal BP^17^. It represents a perforated pendant with a smooth surface (Extended Data Fig. 4). In collaboration with the Württemberg State Museum, the pendant was µCT scanned. It also shows the inclusion of mollusks but is missing limonite concretions and the dissolution of nuclei. Its ooid size distribution is also fundamentally different from the Venus (Fig. 3). The oolite might be of local origin since Jurassic oolites do occur in southern Germany. However, Solnhofen in our sample is an unlikely source for the Brillenhöhle artefact due to the much better sorting and fewer bioclasts in the Brillenhöhle oolite.

## Discussion

Our sampled oolites in this study represent snapshots of oolite-bearing sites. Although our analysis comprises one of the largest oolite thin-section- and µCT-samples published from Europe, it is certainly not complete, especially not for those sites that were exposed on the surface in a time period around 30 ka. Oolites can also be heterogenous within a site. Figure 3 provides a clue for this kind of variation since several sites were represented by multiple samples. Sega di Ala, Isjum A, Isjum B, and Passo delle Fittanze show that there is natural variation in grain size distribution, but in the framework of the whole sample, associated specimens are plotting in the closer vicinity of their counterparts (Fig. 3B). For the archaeological samples Brillenhöhle and Venus from Willendorf we also recognize variation—even in a spatially very restricted object of just a few centimeters. Note, for instance, the small differences between the Venus head (both samplings together) and the Venus leg (four samplings together). Nevertheless, all Venus samples still show the smallest Hellinger distances to each other in our statistics, interrupted only by Sega di Ala (Fig. 3A).

Even if we cannot claim with absolute certainty that the raw material of the Venus originates from a particular locality, the match between the Venus and Sega di Ala samples is almost perfect and suggests a very high probability for the raw material to come from south of the Alps. The Loppio Oolitic Limestone is distributed over an area of approx. 3500 km^2^ on the Early Jurassic Trento Platform in the Southern Alps of Northern Italy^19^. The oolitic facies was deposited during the late Sinemurian transgression and is part of the Calcari Grigi Group. The Loppio Oolite Limestones appear with typical cross stratified strata exhibiting oolites of up to 65 m thickness. The formation is subdivided into a Lower Massive Unit with massive oolitic grainstones and the Upper Stratified Unit with cross-bedded oolitic grainstones. The Loppio Oolitic Limestone and the Venus oolite agree in size distribution of ooids, display a bimodal distribution related to sorting in individual crossbeds, and both rocks contain fragments of bivalves.

Genetic studies^20,21^ indicate that makers of Gravettian tools and art spread from eastern Europe to the West and dominated most of Europe between 33 and 22 ka. In this regard it is interesting that the second highest concordance of an oolite sample with the Venus is found in Isjum A in the East Ukraine, although the Hellinger distances of both samples are clearly larger than for Sega di Ala and the distribution form is different (Fig. 3). The other represented Isjum B site, about 17 km away from Isjum A, is even more disagreeing. Nevertheless, it is worth to mention that there is a clear interchange of ideas in ritual, symbolic and technological behavior between Central Europe and the Russian sites in the area of Kostenki, Gagarino and Avdeevo in the Mid Upper Palaeolithic, which can be demonstrated by the astounding similarity of female figurines, personal ornaments, and lithic technology^22^. In a recent study^23^ using cladistic analysis of 30 discrete stylistic traits of 27 European figurines, a Russian venus cluster could be unmistakably recognized, but the clearly older Venus from Willendorf was closely associated with Russian Kostenki 3, Zaraysk, and several figurines from Avdeevo. Isjum A is only 280 km from Kostenki (Fig. 4), but at least 1600 km airline from Willendorf.

As mentioned, Stránská Skála (Czech Republic) was reported in the literature to represent the potential source of material for the Venus from Willendorf^7^. However, in our study we can exclude this locality as a source. Both the grain size distribution data as well as the claimed absence of biogene inclusions^5,7^ (see Fig. 3, Extended Data Fig. 1) do not match the Willendorf Venus, which is relatively rich in mollusks and shows much smaller ooids than Stránská Skála. Likewise, all other localities in the vicinity of Willendorf (within a radius of 200 km such as Bisamberg, Buschberg, Devin, Dörfles, Ernstbrunn, Großer Torstein, Kettlasbrunn, Kalblsaualm, Mendling) display either very different grain size distributions, sedimentary composition, or represent Miocene oolites. Solnhofen, only ~ 110 km airline southwest from Hohle Fels and Brillenhöhle, shows a very heterogenous composition and too many bioclastic inclusions to be a possible candidate. The other localities in Bosnia-Herzegovina, Crimea, France, Germany, Italy, Romania, Poland, and Switzerland also turned out to be too heterogenous, too young, or too different in grain size distribution. A few sites such as Mezza Selva (northern Italy), Raclawice (Poland) and Santa Massenza (northern Italy) are structurally and in relation to age at least potential candidates, but all of them show larger Hellinger distances for grain size distribution.

**Figure 5:**
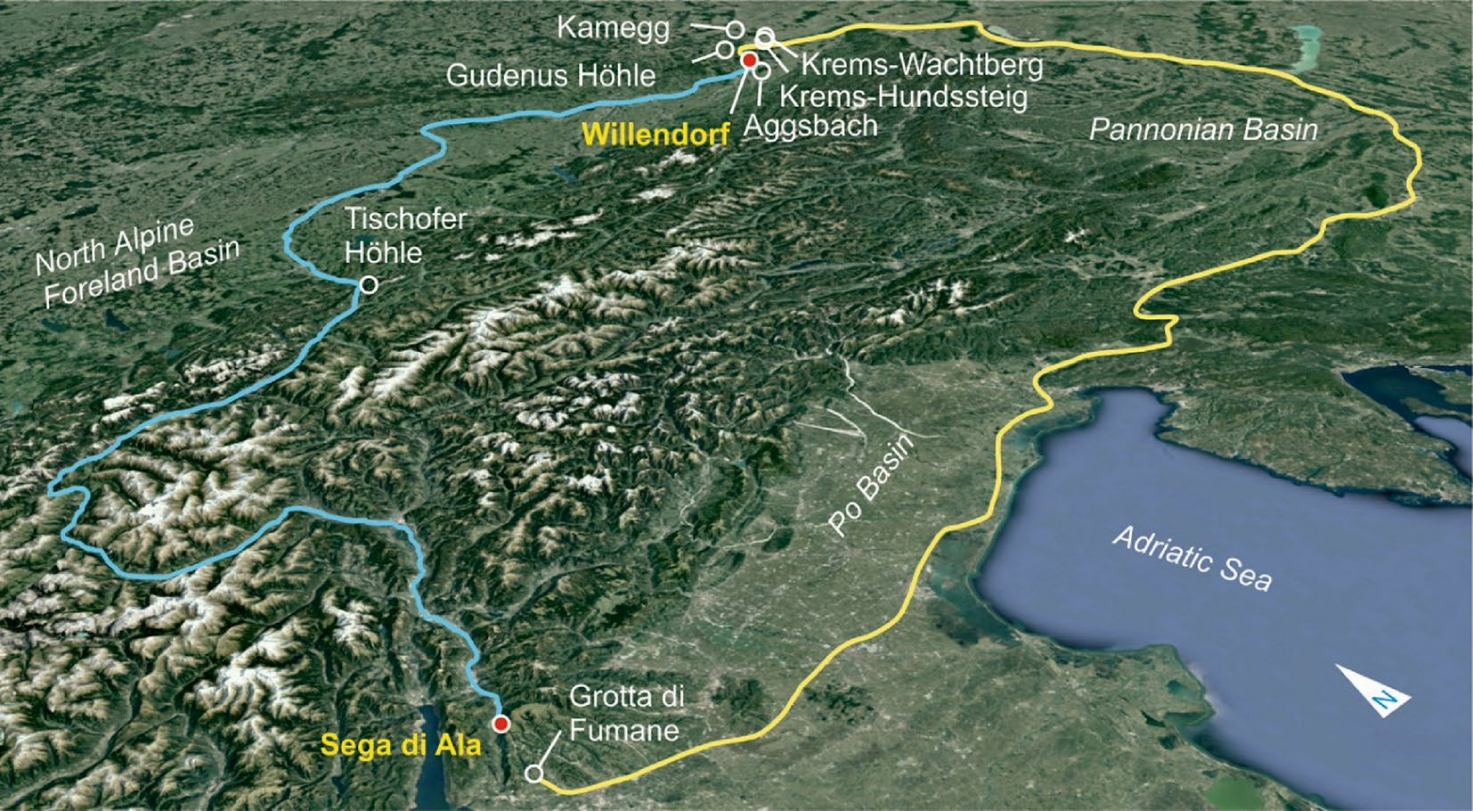
Tentative migration routes from northern Italy to Lower Austria. The yellow path is drawn after the simulations from^25^. The hypothesized blue path from Sega di Ala (northern Italy) to Willendorf (Lower Austria) through the Alps follows the major rivers Etsch, Inn, and Danube. Sega di Ala is located near the important paleolithic site of Grotta di Fumane. Willendorf is located near a cluster of paleolithic sites from different epochs in Lower Austria (e.g., Krems-Hundssteig, Krems-Wachtberg, Aggsbach, Gudenushöhle, Kamegg, Stratzing). Created with Google Earth Data SIO, NOAA, U.S. Navy, NGA, GEBCO, Google Earth Version 7.3, Image Landsat/Copernicus, http://www.earth.google.com [12/14/2015].

What do our results mean with regard to the life and mobility of Gravettian people? The Sega di Ala oolite probes are virtually indistinguishable from the material of the Venus from Willendorf. Sega di Ala is located only 16 km airline from Grotta di Fumane, a prehistoric karstic cave near Lake Garda, which bears findings from the Mousterian, Aurignacian, Uluzzian, and also Gravettian^24^. In GIS-based simulations, two possible optimal-path routes for the migration of Gravettian people between 37 and 30 ka from North to South were found^25^, one leading from Geissenkloesterle (Swabian Jura) south across the Alps to Grotta di Fumane, and the second—bypassing the Alps—from the slightly younger Gravettian site Krems-Hundssteig (Lower Austria, near Willendorf) also in southern direction to Lake Garda^25^ (Fig. 5 yellow). This roughly 930 km long route could also have been used in the reverse direction from South to North. Both directions imply that Gravettian people must have crossed the Danube in Lower Austria, where the river, like today, was considerably broader than upstream in Germany.

The exact time when the Venus was created or its material collected and transported is unknown. However, independent of the location of origin, we can state with certainty that its individual owners kept and protected it en route. A rapid transport from northern Italy to Lower Austria within months would probably have been technically possible, but would rather require a purposeful motivation behind the journey, which seems questionable. The travel of the Venus or its material from northern Italy to the Danube is more likely the outcome of a series of undirected incidents which may have required years or even generations, and, given the possible time range for deposition of the Venus at Willendorf between 30.8 and 29.2 ka cal BP^3^, could have started as early as ~ 31 ka. This coincides with the end of the partly warmer Marine Isotope Stage (MIS) 3, when temperatures decreased further towards the Last Glacial Maximum^26^. Around 31,000 cal. BP, Gravettian people at Hohle Fels left the Ach Valley^27^. Changing vegetation and availability of prey could have forced the hunter-gatherer communities to search for new habitats. Although there are signs of considerable deterioration of the climate at that time, an alternative scenario seems worth exploring. The Alpine glaciers were very dynamic during the last glacial cycle and there is a tendency in studies to overestimate the ice cover in the eastern Alps^28^. A hunter-gatherer group could have crossed the Alps relatively quickly (Fig. 5 blue) if, for instance, subsistence or social conflicts forced them to do so. Following the major river valley of the Etsch and passing Reschensee*,* the broad Inn Valley, and subsequently, the Danube could have been reached. At Passau (Germany), where the confluence of the two rivers is located, the Danube was naturally much narrower than downstream in Lower Austria, offering an easier option for crossing to the north shore. Following the Danube, as their predecessors had done already more than 10,000 years earlier^29^, Willendorf would have been reached easily. Along this roughly 730 km long route between Sega di Ala and Willendorf, the altitude (measured today) is constantly below 1000 m a.s.l., except for a relatively short distance of ~ 35 km at Reschensee*,* which bridges between the Etsch and the Inn with a maximum altitude of 1530 m a.s.l. Still, such a crossing of the Alps remains a more questionable hypothesis while the longer route bypassing the Alps in the East provides a straightforward option for a long-term migration route.

Long distance travel of artefacts during the Gravettian could already be demonstrated in some cases (for a comparison with the Aurignacian see^30^). For instance, the ivory figurines found in the Balzi Rossi caves (northern Italy, Liguria^31^) were possibly transported either along the coast from West or even from northern latitudes, e.g., the Rhone valley. However, as the findings are not stratified, dating is unsecure: they could also originate from the Epigravettian, thus after the Last Glacial Maximum. More evidence comes from Mediterranean molluscs that were found at Sprendlingen near the Rhine in Middle Germany^32^, which is located more than 600 km airline away from the nearest coast of the Mediterranean Sea. At Poiana Cireşului in Romania, perforated Mediterranean shells were discovered in an Early Gravettian layer, documenting transport of at least 900 km across Europe^33^. The Venus I from Willendorf is exceptional in the archaeological record with regard to the material it was made of. This material or the crafted figurine was possibly transported from south of the alpine edge to Willendorf at the Danube, which would suggest a diffusion of human groups bypassing, or even crossing, the Alps in the times before the Last Glacial Maximum. While this is the most likely result from our analysis, it cannot be ruled out, although based on a lower statistical likelihood, that the material or the crafted figurine could originate from the area of the eastern Ukraine, which would indicate a long-term and long-distance diffusion of cultural artefacts over generations from the East to the West^34^. In any event, our results suggest considerable mobility of Gravettian people in the time around 30,000 years ago.

## Methods

### Acquisition of oolite samples

Oolites are peculiar rocks and their occurrences are accurately documented on geological maps and frequently described in the literature. We have screened the literature for such oolite occurrences in continental Europe and collected multiple samples from 18 localities in Austria, the Czech Republic, Poland, Romania, Croatia, Bosnia and Herzegovina, Italy and Russia by own fieldwork and from utilizing the collections of the Natural History Museum Vienna (Austria). In addition, we acquired samples from 15 localities in France, Switzerland, Germany, Poland and the Ukraine from regional geologists.

### Rationale for further sample selection

No excluding criteria were applied a priori regarding our oolite sampling strategy. All oolites were analyzed in respect to their texture (size, sorting and density of ooids) and fossil content (size, quantity, taxonomic composition, fragmentation). Later, the Venus-oolite was biostratigraphically dated by us as Jurassic. Therefore, all older (Triassic) and all younger (Cretaceous, Tertiary) samples were excluded from further statistical analyses. Whilst fossils are floating in low numbers among the ooids in the Venus, many oolite samples display a very heterogeneous texture of ooids and bioclasts, i.e. fragments of fossils. Therefore, samples in which bioclasts occur in rock-forming amounts and/or showing very different large size were also excluded from analyses. Extended Data Table 4 summarizes these properties. Note that excluding criteria are listed exemplarily and not exhaustively.

### µCT scanning

A first scan of the Venus from Willendorf was undertaken on January 8th 2013 at the Core Facility for Micro-Computed Tomography at the University of Vienna with a custom built VISCOM X8060 (Germany) μCT scanner: 150 kV, 210 μA, 1400 ms, diamond high performance transmission target, 0.75 mm copper filter, spatial resolution 53 μm. Further scans were undertaken in the same lab on March 22nd 2016, focusing on the leg and the head regions: 150 kV, 150μA, 1400 ms, diamond high performance transmission target, 0.50 mm copper filter, spatial resolution 11.5 µm. X-ray images were taken from 1440 different angles. Using filtered back-projection in VISCOM XVR-CT 1.07 software, these data were reconstructed as 3D volumes with a color depth of 16,384 grey values. For work on the gross morphology of the whole figurine and on limonites, the 53 µm-scans were used. For extracting the bivalve, 11.5 µm-scans of the head were used. For grain size analysis, six individual 11.5 µm-slices were sampled: head region: xy-slice 800, xy-slice 1600; leg region: xy-slice 650, xy-slice 700, xy-slice 1100, xy-slice 1300.

The Brillenhöhle pendant was scanned on March 8th 2017 at the Core Facility for Micro-Computed Tomography at the University of Vienna with the same scanner using the following parameters: 150 kV, 150μA, 1901 ms, diamond high performance transmission target, 0.50 mm copper filter, spatial resolution 11.5 µm.

Individual oolite samples were also µCT scanned prior to sectioning between 2016 and 2018 at the Core Facility for Micro-Computed Tomography at the University of Vienna with similar settings as the Venus and the pendant, which varied only slightly in exposition time: 150 kV, 150 μA, 1900–2500 ms, diamond high performance transmission target, 0.75 mm copper filter, spatial resolution 11.5 µm.

### Thin sectioning

Lithological analysis of thin sections made from oolitic limestones provides unique information regarding the provenience of the sample, fossil or archaeologic figurine. Grain size and sorting of particles (ooids) are most important for the comparison with the µCT scans of the Venus. Coated grains such as ooids exhibit an external cortex with a concentric structure of aragonite, calcite or micrite and an internal nucleus (e.g. fossils, lithoclasts or minerals).

Preparation for thin sectioning included the following steps: sampling larger rocks (in field and collections), cutting of a quadratic rock sample of approx. 40 × 40 × 10 mm, grinding the rock slice to complete planeness by using SICA powder (silicium carbide with grain size 220, 600 and 1000 ym), using one-side sandblasted glasses (50 × 50 mm) with complete planeness and a defined thickness (1.55 mm), preparation of the rock slice surface with a cast resin “water-clear” UN 3082 and hardener “water-clear” UN 2735, fixing the rock slice with the resin ARALDITE AY 103-1 and hardener ARADUR HY 951 to the sand blasted glass slide, cutting off rock material to a thickness of 0.3 mm, grinding of thin sections to a thickness of 60 µm, finally grinding the thin sections by using SICA powder (silicium carbide with grain size 220, 600 and 1000 ym) to a final thickness of the thin section of 20 µm (+ resin).

Digital high-quality photomicrographs of the thin sections were performed on a Discovery.V20 Stereo Zeiss microscope. Specific magnifications were × 4.7, × 10.5 and × 40 in transmitted light mode. Images from the AxioCam MRc5 Zeiss were processed and documented by using the AxioVision SE64 Rel. 4.9 imaging system. Additional photomicrographs were made from surfaces of the rock materials which were the basis for the thin section analyses. New surface photomicrographs of the Venus figurine were conducted in incident light mode.

### Segmentation and measurements of inclusions

The µCT image stacks were imported in Amira 6.5. (ThermoFisher Scientific) and virtually segmented to isolate the limonite concretions and individual biogene inclusions such as the bivalve described above. We applied a semi-automatic segmentation algorithm based on the half-maximum-height value protocol proposed by Spoor and colleagues^35^. Afterwards, we generated 3D virtual surface models for limonite concretions and the bivalve shell. The limonite concretions were then measured along the three major axes of the ellipsoids. A virtual surface model of the Venus was also generated and the size of hemispherical cavities measured in two dimensions (the third dimension was not applicable because the cavities were open). Means were calculated in IBM SPSS Statistics 27 (Extended Data Table 2).

### Grain size distribution

µCT (CT) and thin-section (TS) samples were edited and grain sizes measured using the software ImageJ^36^. In the case of the TS images, pixel size was specified by measuring the scales drawn on the thin sections (the resulting distance in pixels equaling 1 µm in the scans). For the CT images, pixel size was known from the scan parameters (11.5 µm).

To edit the depicted objects for measuring, the samples were first adjusted in terms of brightness and contrast, after which they were converted to binary (black and white images). In cases where it was necessary to decrease noise, the functions “despeckle”, “median” (filter) and “remove outliers” were applied, all of which are median filters that replace pixels with the color values of their surrounding pixels^37^. When removing outliers, default values (radius: 2.0 px, threshold: 50) were used. Individual objects were then further corrected by hand (pencil tool and paintbrush tool), e.g., to over-paint white pixels or trace ill-defined objects, by visual comparison with the original images. To isolate individual objects, the function “Watershed” was used, which automatically separates objects with white lines. This segmentation method calculates Euclidian distance maps, wherein the ultimate eroded points (local maxima) are found and then dilated until the edge of the object or another dilating line is reached^37^. The resulting separations were corrected with the pencil tool, again by comparison with the original images.

The resulting objects were then analyzed, excluding the objects at the edges. Clearly identifiable ooids were selected, while lithoclasts, biogenic materials, and other parts of the matrix, as well as ill-defined ooids (e.g., parts of very dense accumulations), were de-selected. The generated selections were measured, resulting in a Feret’s diameter for each ooid. All further analyses were conducted with these diameters, which constitute the longest distance between any two points along each selection boundary^37^.

### Statistics

All grain diameters were log transformed (natural logarithm), and for each site the probability density function (pdf) was computed by smoothed kernel density estimation with Silverman's rule to determine bandwidth. For quantifying the overall difference between the size distributions of two sites, we used the Hellinger distance, *H*, between the corresponding probability density functions:

$$H^{2} (f_{i} ,f_{j} ) = 1 - \int {\sqrt {f_{i} (x)f_{j} (x)} } dx,$$

where *f*_*i*_ and *f*_*j*_ are the pdfs for sites *i* and *j*, and *x* is the log-transformed diameter (e.g.,^38^). Hellinger distance is a metric distance function and measures the overlap of two distributions, where *H* = 0 indicates identical distributions and *H* = 1 no overlap at all. The value 1 − *H*^2^, also referred to as Bhattacharyya coefficient, relates to the mean (root) likelihood of the elements of sample *i* to be drawn from sample *j* (and vice versa). We computed the Hellinger distances between each site and the pooled Venus sample (comprising all head and leg samples).

Furthermore, we performed an ordination analysis (principal coordinate analysis, also called metric multidimensional scaling^39^) of all the pairwise Hellinger distances between the sites. The pairwise Euclidean distances in the plot of the first two principal coordinates accounted for 76% of the variance among the full Hellinger distances, and the correlation between pairwise Hellinger distances and the pairwise Euclidean distances within the first three principal coordinates was *r* = 0.97, indicating a good representation.

### Comparison of µCT and thin-sections

For all oolite localities, thin sections could be produced and used to determine grain size distribution. For the Venus from Willendorf and the Brillenhöhle pendant, naturally no thin sections were available and µCT images had to be used. We scanned some of the other oolite samples before thin sectioning to evaluate potential differences in the grain size distribution between thin sections and µCT. Thin sections have a slightly higher spatial resolution, but for a µCT scan resolution of 11.5 µm, this is of lesser importance for measuring the diameters of particles with a typical size range of 0.15 mm to 1.5 mm. Thin sections offer a better contrast than µCT images. Due to the x-ray absorption characteristics of the calcified ooids, the cortex, the biogene inclusions, and the ooid-nuclei, these images delivered varying qualities, ranging from a good representation of ooids to a very poor differentiation, which made it practically impossible to delineate ooid borders. The Venus as well as the Brillenhöhle pendant showed relatively good differentiation. For some oolite localities offering a relatively good differentiation (Savonnieres, Stránská Skála) and an experimental sample from the Bahamas including only grains, we measured grain size distribution in thin-sections as well as in µCT scans. As Extended Data Fig. 5 shows, for the experimental sample of grains from the Bahamas, which is not disturbed by any inclusions, it is almost a perfect match. In the case of oolites, the distributions are largely overlapping, but µCT slightly overestimates the grain size because smaller ooid-sections tend to become overlooked due to the poorer differentiation. As the Venus grains are among the smallest in our sample, such an overestimation would not change our results since the other samples with smaller grain size are Sega di Ala and Isjum. We therefore conclude that data from thin-sections and µCT can be combined for this analysis.

## Supplementary Information

Supplementary Information.

## Data Availability

Raw data related to the Venus from Willendorf are available from the corresponding author upon request. 3D surface data will be made available upon publication in the “digital@rchive of fossil hominoids” (https://www.virtual-anthropology.com/3d-data/data-webshop/) and on a server of the Natural History Museum Vienna. Oolite data are available upon request from the Natural History Museum Vienna.
