# Supplementary material for: The microstructure and the origin of the Venus from Willendorf
Source: Sci Rep. 2022 Feb 28;12:2926. doi: 10.1038/s41598-022-06799-z (PMC8885675; doi:10.1038/s41598-022-06799-z)

Supplementary Information

"The microstructure and the origin of the Venus from Willendorf"

Authors:

Gerhard W. Weber[1,7]†*, Alexander Lukeneder[2]†, Mathias Harzhauser[2]†, Philipp Mitteroecker[3], Lisa Wurm[4], Lisa-Maria Hollaus[1,7], Sarah Kainz[1,7], Fabian Haack[5], Walpurga Antl-Weiser[6], Anton Kern[6]

Corresponding author details:

Gerhard W. Weber
University of Vienna, Department of Evolutionary Anthropology
Althanstrasse 14, A-1090 Vienna, Austria
gerhard.weber@univie.ac.at
+43-1-4277-54701

Extended Data Table 1. Oolite localities in the sample

Oolite localities

Locality	Country	Coordinates	Period	Age	Sample code
Arshinzevo/Kertsch	Crimea	45°17'04.5''N 36°24'56.2''E	Pliocene	Kimmerian	Arshinzov.a
Aschersleben	Germany	51°45'26.19"N 11°27'42.55"E	Triassic	Olenekian	Ascher.CT.area
Bahamas	Bahamas	25° 2'4.80"N 77°23'40.08"W	Quaternary	Holocene	Bahamas.a.
Bella Lasta	Italy	46° 2'35.05"N 11° 7'32.55"E	Jurassic	Sinemurian	BL.54.area
Bisamberg	Austria	48°19'53.78"N 16°21'41.47"E	Jurassic	Tithonian	Bisamberg.a
Brauvilliers	France	48°35'4.86"N 5° 8'36.69"E	Jurassic	Tithonian	B-01.a
Brillenhöhle	Germany	48°24'19.45''N 9°46'38.9''E	Jurassic	Tithonian	Brillenhöhle.a
Buschberg	Austria	48°34'42.35"N 16°24'31.00"E	Jurassic	Tithonian	Busch.0030.area
Devin	Slovakia	48°10'35.79"N 16°59'2.53"E	Jurassic	Tithonian	Devin.a
Dörfles	Austria	48°32'30.15"N 16°21'8.29"E	Jurassic	Tithonian	Dörfles.a
Ernstbrunn	Austria	48°31'33.56"N 16°21'33.92"E	Jurassic	Tithonian	Ernstbrunn.a
Faro San Croce	Italy/Sicily	37°14'36.60"N 15°15'21.72"E	Miocene	Messinian	FaroSanCroce.a
Grabovice	Bosnia-Herzegovina	44°29'2.52"N 17°30'27.71"E	Jurassic	?Tithonian	Grabovice.a
Großer Torstein	Austria	47°39'58.71"N 14°54'59.78"E	Triassic	Rhaetian	Gr.Torstein.a
Iasi	Romania	47° 9'20.9"N 27°36'4.6"E	Miocene	Sarmatian	Iasi.a.
Isjum A	Ukraine East	49°13'28.21"N 37° 6'14.26"E	Jurassic	Tithonian	Isjum.a, Nerinea
Isjum B	Ukraine East	49° 8'4.80"N 37°17'40.25"E	Jurassic	Tithonian	Isjum1&3
Kalbslaualm	Austria	47°45'19.05"N 15°48'12.40"E	Triassic	Rhaetian	Kalblsaualm.a
Kettlasbrunn	Austria	48°32'32.4"N 16°30'01.3"E	Miocene	Sarmatian	Ket.a
Małogoszcz	Poland	50°49'17.09"N 20°15'34.33"E	Jurassic	Kimmeridgian	Malagosz.CT.a
Mali Kamishlack	Crimea	45°26'18.5''N 36°30'35.3''	Miocene	Tschokrakian	Mali.a
Mendling	Austria	47°44'18.48"N 14°52'55.41"E	Triassic	Carnan	Mendling.a
Mezza Selva	Italy	45°52'14.43" N11°25'29.49"E	Jurassic	Sinemurian	MS21.area
Montricher	Switzeland	46°36'11.8"N 6°23'57.9"E	Cretaceous	Hauterivian	Hauterian.a
Morley	France	48°34'37.77"N 5°14'46.77"E	Jurassic	Tithonain	M329.a
Passo delle Fittanze	Italy	45°41'02.91"N 10°58'44.24"E	Jurassic	Sinemurian	Passeo.a.
Racławice	Poland	50°19'20"N 20°14'12"E	Jurassic	Callovian	Racla.area
Santa Massenza	Italy	46° 4'13.78"N 10°58'51.28"E	Jurassic	Sinemurian	A1.CT.area
Savonnières	France	48°35'48.20"N 5° 7'52.81"E	Jurassic	Tithonanian	Savonnieres.a
Sega di Ala	Italy	45°43'40.65"N 10°58'1.71"E	Jurassic	Sinemur	ISA3.CT.area
Sjedinovac	Bosnia-Herzegovina	44° 6'8.33"N 16°27'38.80"E	Triassic	Norian/Carnian	Sedinovac.a
Solnhofen	Germany	48° 53' 29.70"N 10° 58' 37.83"E	Jurassic	Tithonanian	Solnhofen.CT.a
Stránská skála	Czech Republic	49°11'17.61"N 16°40'35.09"E	Jurassic	Tithonanian	Stranska.a.
Tesero	Italy	46°17'21.67"N 11°30'28.49"E	Triassic	Induanian/Olenekian	Tesero.a
Venus	Austria	48°19’23.50’’N, 15°24’15.20’’E	Pleistocene	late Pleistocene	Venus.K700.a

Extended Data Table 2. Size of embedded limonite concretions and hemispherical cavities on the Venus surface

**Limonites (see Figure 2)**	**Diameter 1**	**Diameter 2**	**Diameter 3**	**Mean Diameter**	**Unit**
orange	2,85	3,21	3,12	3,06	mm
blue	2,48	2,54	2,59	2,54	mm
red	2,22	2,05	2,45	2,24	mm
yellow	3,09	3,03	3,23	3,12	mm
green	2,68	2,79	2,53	2,67	mm
purple	3,07	3,02	2,84	2,98	mm
**Grand Mean**				**2,77**	**mm**
**Standard Deviation**				**0,34**	**mm**

**Hemispherical cavities on the Venus surface**	**Diameter 1**	**Diameter 2**	**Diameter 3**	**Mean Diameter**	**Unit**
right knee region lateral	2,76	2,13	*n/a	2,45	mm
right hip lateral	3,47	3,79	*n/a	3,63	mm
navel	2,97	2,13	*n/a	2,55	mm
right breast	1,84	2,06	*n/a	1,95	mm
cleavage	3,63	3,71	*n/a	3,67	mm
left thigh posterior	2,95	3,08	*n/a	3,02	mm
face	1,53	1,43	*n/a	1,48	mm
left upper arm posterior	1,90	1,79	*n/a	1,85	mm
**Grand Mean**				**2,57**	**mm**
**Standard Deviation**				**0,81**	**mm**

Mann-Whitney-Test for difference of size Z=-0.645 p=0.573

*n/a: the third dimension could not be measured on the hemispherical cavities

Extended Data Table 3. Hellinger distances of samples to the Venus average

**Sample**	**H**
Venus pooled	0,0000
Venus leg	0,0303
Venus leg	0,0463
Venus leg	0,0477
Sega di Ala	0,0867
Venus leg	0,0868
Venus head	0,1576
Venus head	0,1585
Sega di Ala	0,1971
Isjum A	0,2319
Isjum A	0,2681
Mezza Selva	0,3004
Raclawice	0,3157
Isjum B	0,3186
Santa Massenza	0,3451
Isjum B	0,3757
Ernstbrunn	0,4251
Grabovice	0,4853
Buschberg	0,5277
Brauvilliers	0,5527
Malogoszcz	0,5676
Bella Lasta	0,5733
Savonnieres	0,6022
Stranska Skala	0,6252
Morley	0,6572
Passo delle Fittanze	0,6585
Brillenhöhle	0,6646
Brillenhöhle	0,6726
Brillenhöhle	0,6846
Passo delle Fittanze	0,6870
Devin	0,7667

Extended Data Table 4. Properties of the 33 oolite samples in the study. Those 15 samples that could be excluded based on stratigraphy or composition are marked in grey. The 18 remaining samples kept for statistical analysis of grain size distribution are marked in green.

Oolite localities sampled

					**Excluded from grain size anal. based on**	
**No.**	**Locality**	**Country**	**Period**	**Age**	**Stratigraphy**	**Bioclasts**
1	Arshinzevo/Kertsch	Crimea	Pliocene	Kimmerian	too young	
2	Aschersleben	Germany	Triassic	Olenekian	too old	
3	Bella Lasta	Italy	Jurassic	Sinemurian		
4	Bisamberg	Austria	Jurassic	Tithonian		too many, too large
5	Brauvilliers	France	Jurassic	Tithonian		
6	Brillenhöhle	Germany	Jurassic	Tithonian		
7	Buschberg	Austria	Jurassic	Tithonian		
8	Devin	Slovakia	Jurassic	Tithonian		
9	Dörfles	Austria	Jurassic	Tithonian		too many, too diverse
10	Ernstbrunn	Austria	Jurassic	Tithonian		
11	Faro San Croce	Italy/Sicily	Miocene	Messinian	too young	
12	Grabovice	Bosnia-Herzegovina	Jurassic	?Tithonian		
13	Großer Torstein	Austria	Triassic	Rhaetian	too old	
14	Iasi	Romania	Miocene	Sarmatian	too young	
15	Isjum A	Ukraine East	Jurassic	Tithonian		
16	Isjum B	Ukraine East	Jurassic	Tithonian		
17	Kalbslaualm	Austria	Triassic	Rhaetian	too old	
18	Kettlasbrunn	Austria	Miocene	Sarmatian	too young	
19	Małogoszcz	Poland	Jurassic	Kimmeridgian		
20	Mali Kamishlack	Crimea	Miocene	Tschokrakian	too young	
21	Mendling	Austria	Triassic	Carnian	too old	
22	Mezza Selva	Italy	Jurassic	Sinemurian		
23	Montricher	Switzeland	Cretaceous	Hauterivian	too young	
24	Morley	France	Jurassic	Tithonain		
25	Passo delle Fittanze	Italy	Jurassic	Sinemurian		
26	Racławice	Poland	Jurassic	Callovian		
27	Santa Massenza	Italy	Jurassic	Sinemurian		
28	Savonnières	France	Jurassic	Tithonanian		
29	Sega di Ala	Italy	Jurassic	Sinemurian		
30	Sjedinovac	Bosnia-Herzegovina	Triassic	Norian/Carnian	too old	
31	Solnhofen	Germany	Jurassic	Tithonanian		too many, too diverse
32	Stránská skála	Czech Republic	Jurassic	Tithonanian		
33	Tesero	Italy	Triassic	Induanian/Olenekian	too old	

Extended Data Figure 1. µCT slice of the Venus head 1600, Venus right leg 700 and Brillenhöhle pendant; thin sections of Sega di Ala, Isjum A, Stránská Skála, Santa Massenza, and Raclawice; all images scaled to same scale, 1000µm bar provided.

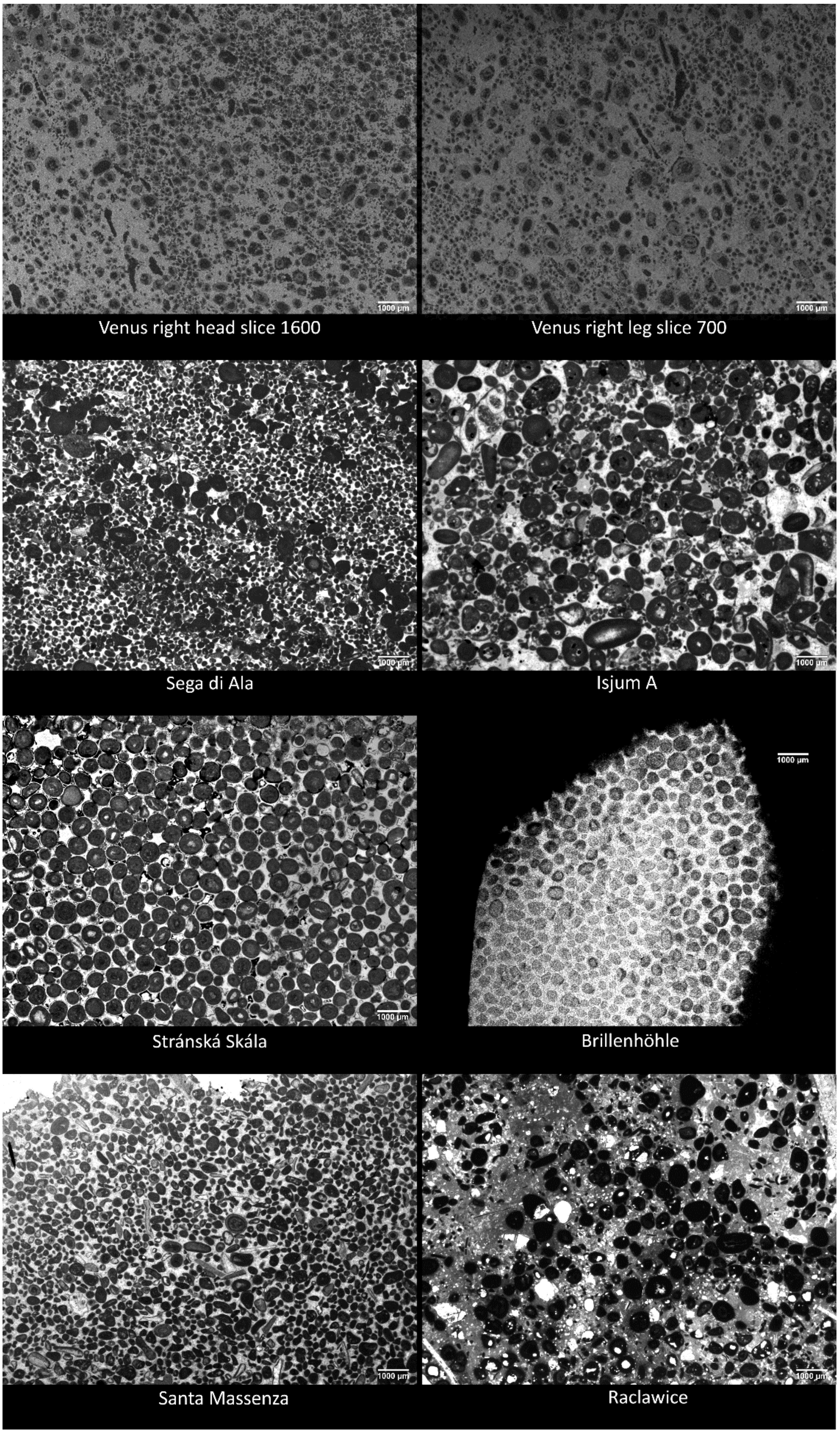

Extended Data Figure 2. Representatives of the bivalve families Pteriidae (1: Pteria, recent, 1x), Oxytomidae (2: Oxytoma, Jurassic, 2x) and Bakevelliidae (3: Phelopteria, Cretaceous, 1x) (modified from [14]).

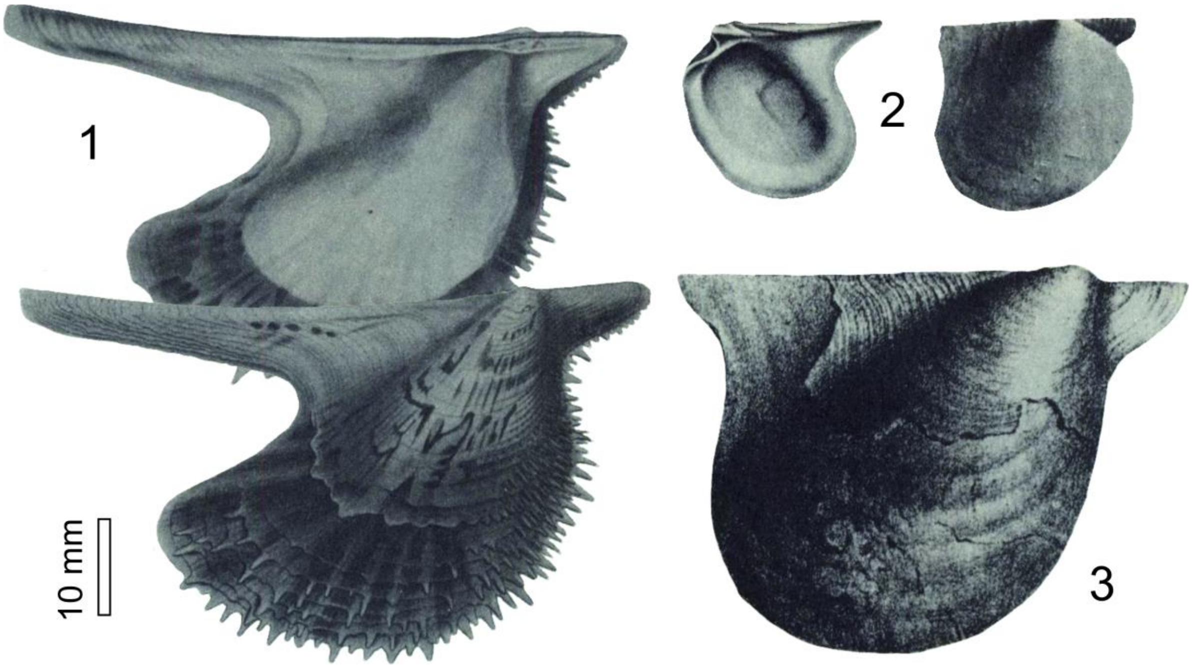

Extended Data Figure 3. Surface of the Venus captured by reflected-light microscopy;

A: semispherical cavity formed by broken-off limonite concretion at transition from right thigh to hip;

B: detail showing the red ochre partly filling the hollow nuclei of the ooids; C: closer zoom of A.

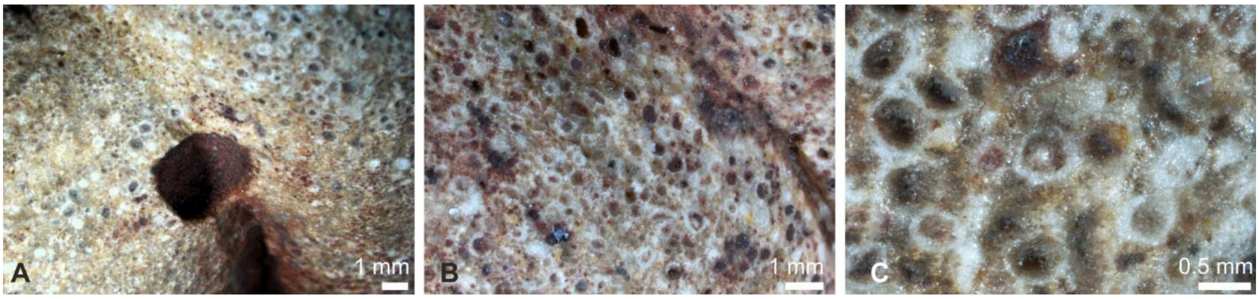

Extended Data Figure 4. Original Brillenhöhle pendant (left) and reflected-light microscopy images x4.7 (right)

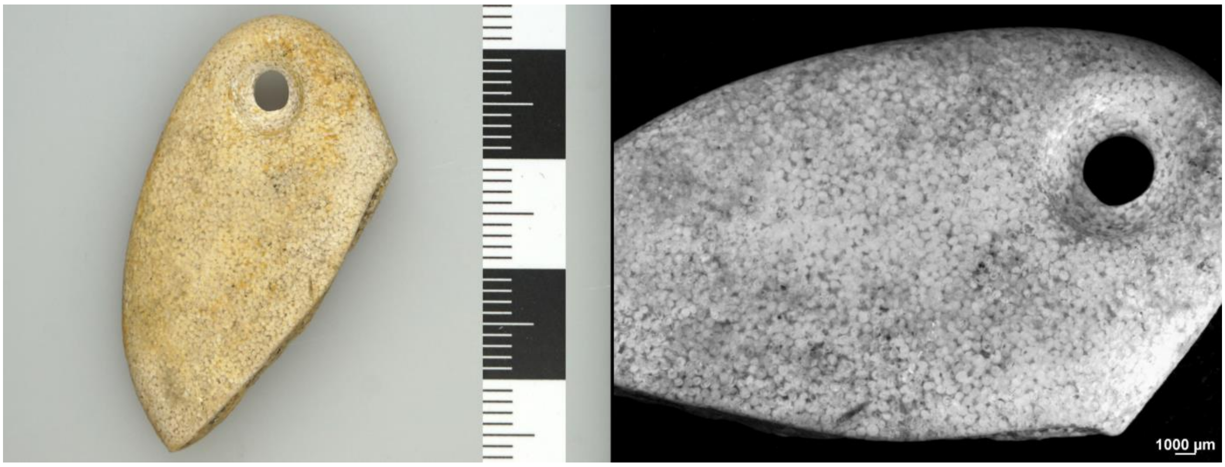

Extended Data Figure 5. Comparison of grain size distribution measured from thin-sections and µCT-scans. The Bahama sample is an experimental one that does not contain any matrix or inclusions. The match between measurements is perfect. For the two oolite sample from Savonnieres and Stránská Skála the match is good but shows a slight overestimation of grain size for µCT.

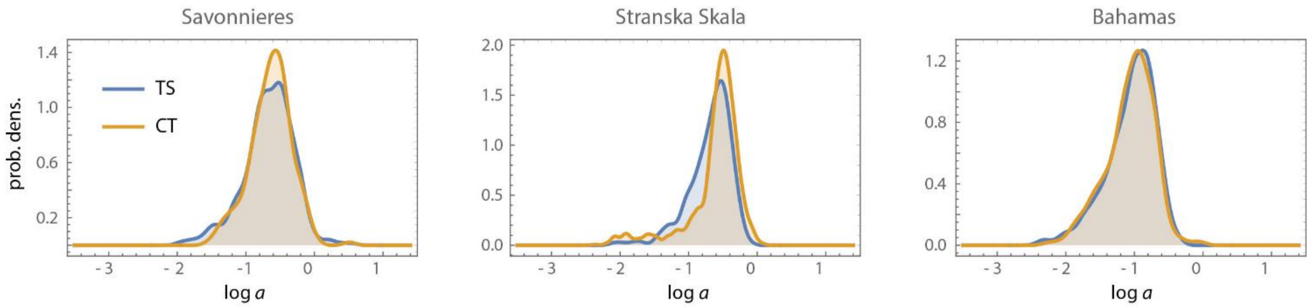

Supplement: Supplementary file 1 — Supplementary Information. [file 41598_2022_6799_MOESM1_ESM.pdf]
